# Periodontal clinical evaluation before and after surgically assisted rapid maxillary expansion

**DOI:** 10.1590/2177-6709.23.1.079-086.oar

**Published:** 2018

**Authors:** Michelle Sendyk, Wilson Roberto Sendyk, Débora Pallos, Letícia Cristina Cidreira Boaro, João Batista de Paiva, José Rino

**Affiliations:** 1 Universidade de São Paulo, Departamento de Ortodontia (São Paulo/SP, Brazil).; 2 Universidade Santo Amaro, Departamento de Periodontia e Implantodontia (São Paulo/SP, Brazil).

**Keywords:** Periodontics, Orthodontics, Maxillary expansion

## Abstract

**Introduction:**

The surgically assisted rapid maxillary expansion is a procedure that reduces the resistance of the sutures correcting the posterior crossbite in adults.

**Objective:**

The aim of this study was to evaluate the periodontal status of 17 adults submitted to this procedure.

**Methods:**

The clinical attachment level (CAL), gingival recession, attached gingiva and bleeding were evaluated in maxillary first premolars and molars, central and lateral incisors of right and left sides before surgery, 5 days and 6 months after. Means, standard deviation, medians, minimum and maximum values were compared among the evaluations using the Friedman and McNemar tests.

**Results:**

There was a statistically significant increase in CAL in the right central incisor, right and left premolars and right and left molars. There was a statistically significant increase in gingival recession in the right and left premolars and molars. The amount of attached gingiva significantly decreased in right premolars and right and left molars. There was increase in bleeding in most of the teeth.

**Conclusion:**

Results indicated that the surgically assisted rapid maxillary expansion might cause alterations in periodontal tissue.

## RESUMO

**Introdução:**

a expansão rápida da maxila assistida cirurgicamente (ERMAC) é um procedimento que reduz a resistência das suturas, corrigindo a mordida cruzada posterior em adultos.

**Objetivo:**

o objetivo deste estudo foi avaliar o *status* periodontal de 17 adultos submetidos a esse procedimento.

**Métodos:**

o nível clínico de inserção (NCI), a recessão gengival, gengiva inserida e sangramento foram avaliados nos primeiros pré-molares, molares, incisivos centrais e laterais superiores dos lados direito e esquerdo antes da cirurgia, e depois de 5 dias e 6 meses. Médias, desvios-padrão, medianas, valores mínimos e máximos foram comparados entre as avaliações, usando os testes de Friedman e McNemar.

**Resultados:**

houve aumento estatisticamente significativo no NCI no incisivo central direito, pré-molares direito e esquerdo e molares direito e esquerdo. Houve aumento estatisticamente significativo na recessão gengival nos pré-molares e nos molares direito e esquerdo. A quantidade de gengiva inserida diminuiu significativamente nos pré-molares direitos e molares direitos e esquerdos. Houve aumento no sangramento na maioria dos dentes.

**Conclusão:**

os resultados indicaram que a ERMAC pode causar alterações no tecido periodontal.

## INTRODUCTION AND LITERATURE REVIEW

Rapid maxillary expansion (RME) is a procedure used in the treatment of young patients with maxillary atresia. In adults, this procedure has high failure rates due to increased rigidity of the maxillary sutures, and it can cause dental inclinations, osseous dehiscence and gingival recession.^1^ ^-^ ^4^ For this reason, the surgical separation of the midpalatal suture has to be performed. This procedure named surgically assisted rapid maxillary expansion (SARME) is indicated^5^ ^-^ ^7^ in adult patients to correct significant transversal maxillary atresia, posterior crossbite, failure of orthopedic expansion and reduction of buccal corridors of smile.^8^ ^,^ ^9^

The SARME technique can be performed to promote maximum mobility of the maxilla^10^ with higher risk of complications or by a less invasive technique, with a minor risk of complication, but a higher risk of relapse, periodontal problems and unexpected fractures. Some authors^11^ ^,^ ^12^ ^,^ ^13^ associated the subtotal Le Fort I osteotomy with the separation of the maxillary tuberosity from the pterygoid plateau and the osteotomy of the anterior region of the maxilla. However, other authors^3^ ^,^ ^8^ ^,^ ^14^ ^,^ ^15^ did not perform the osteotomy separating the maxillary tuberosity from the pterygoid plateaus, but instead performed the osteotomy in the midpalatal suture. More conservative techniques are described in the literature, without osteotomy of the midpalatal suture and of the pterygomaxillary suture.^6^

Long-term periodontal health is related to the buccal inclination of the anchor teeth of the expansion appliance and to the periodontal condition of teeth after treatment. Excessive buccal inclination of the posterior teeth leads to the formation of osseous dehiscence, contributing to gingival recession.^9^ ^,^ ^16^ The root proximity in the interdental osteotomy can cause periodontal defects. During the SARME, if the resistance to opening the midpalatal suture is very strong, the fracture may not occur symmetrically. After the surgery, periapical and occlusal radiographs must be taken for evaluation of the fracture line. The central incisors should be carefully probed and their pocket depth, compared to the initial values.^17^

Due to the frequent use of the SARME in the correction of transverse skeletal discrepancies in adults and the scarce data in the literature regarding possible periodontal alterations caused by this procedure, the present study aimed to investigate the periodontal status of patients after appliance installation, at the fifth day and six months after surgery.

## MATERIAL AND METHODS

The sample comprised 17 nonsmoking adult subjects (8 male, 9 female), Caucasian, right-handed, with ages from 25 to 45 years old. Based on clinical and radiographic evaluation, patients were diagnosed with maxillary transversal deficiency and SARME was indicated. No patient presented signs and symptoms of spontaneous gingival bleeding, mobility, pathologic migration, pain or sensitivity in any tooth. The exclusion criteria were any health problem that presented a contraindication to surgery.

**Figure 1:**
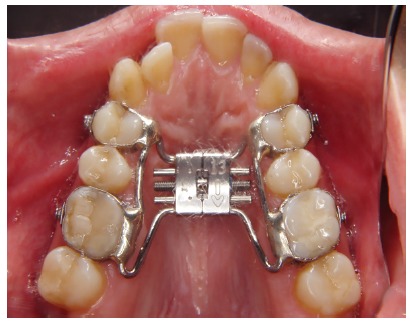
Intraoral occlusal view showing the bonded expansion appliance.

The Research Ethics Committee, Faculty of Dentistry, University of São Paulo approved this study (protocol # 176/2007). Prior to the beginning of the orthodontic treatment, all the patients were submitted to periodontal scaling and root planning and oriented and motivated about effective plaque control and proper oral hygiene control. A Biederman expander appliance with a 13-mm Hyrax screw was manufactured and cemented to the first premolars and molars one week prior to the surgical procedure (Fig 1).

Before the surgery and after the appliance installation (T_0_), the following parameters were evaluated in the maxillary central and lateral incisors, first premolars, first molars, of both sides, in each patient by the same periodontist using a Michigan periodontal probe with Williams markings. This was called initial time (T_0_) and was considered as control.

» Clinical attachment level (CAL): was obtained by measuring the distance from the cemento-enamel junction (CEJ) to the gingival margin, and adding the measure of probing depth in the buccal area.
» Amount of gingival recession: determined by the distance from the cement-enamel junction to the gingival margin.
» Bleeding index: detected after penetration of the probe into the gingival sulcus.
» Amount of attached gingiva (width of keratinized tissue): determined by subtracting the depth of the pocket from the total height of gingiva to the mucogingival junction.

**Figure 2:**
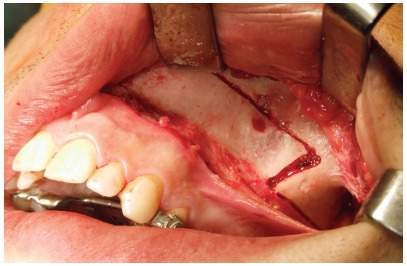
Horizontal osteotomy performed in the lateral wall of the maxilla.

**Figure 3:**
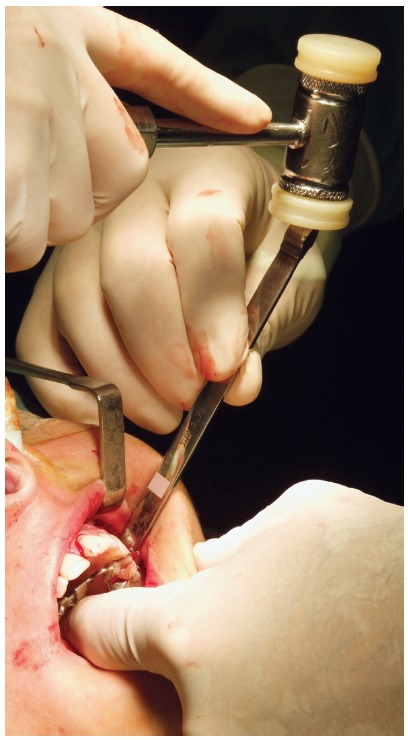
Osteotomy in the pterygoid process.

In these patients, the surgical procedure was performed as a subtotal Le Fort I osteotomy, with separation of the maxillary tuberosity from the pterygoid plateau, associated with the osteotomy of the anterior region of the maxilla^18^ (Figs 2 and 3). An occlusal radiograph was obtained, to check the presence of the fracture line of the alveolar bone mesial to the central incisors. After a latency period of 5 days, the patients began activations and were re-evaluated (T_1_) regarding the above-mentioned parameters. The protocol of activation was 1/4th turn in the morning and 1/4th turn at night, completed by the patient, daily, until the desired expansion was obtained.

The periodontal evaluation was repeated six months after the SARME (T_2_). The same operator performed all periodontal assessments in each of the three stages. For recording all parameters, the examiner was blinded to the previous scores. During the six-month period a periodontal examination was made every fifteen days regarding plaque control motivation.

## STATISTICAL METHODS

The measurements of clinical attachment level, gingival recession, bleeding, and attached gingiva were performed at three distinct times: after the appliance installation (T_0_), at the fifth day after surgery (T_1_) and 6 months after surgery (T_2_) for each tooth. For the probing, the average value of the buccal area was calculated in the three moments; and for the bleeding, the average value of the buccal and palatal areas was calculated in the three moments.

For each variable, means and standard deviations, values were calculated. For comparison of CAL, gingival recession and attached gingiva levels, the Friedman^18^ test was used, and for the tooth with statistically significant differences, nonparametric multiple comparisons for repeated measures were performed to verify differences among the three different times studied.

The bleeding variable was described for each tooth using absolute and relative frequencies, and McNemar test was used to verify differences among the three different study times. For all tests the global significance level adopted was 5%.

For the sample of the present study, the power of the test was 0.98, considering an overall level of significance of 5%. Despite being 17 patients, several measurements were made on the same tooth, which increased the power of the test, resulting in a relatively high value.

## RESULTS

There was a significant increase in the CAL among the evaluations for the right central incisor, the left and right premolar, and left and right molar (*p*< 0.05). The CAL increased significantly from T_0_ to T_1_ and from T_0_ to T_2_ in all teeth mentioned above. However, from T_1_ to T_2_ the increase was significant only in the right central incisor and left premolar (*p*< 0.05) (Table 1).

**Table 1:** Evaluation of mean and standard deviation of CAL of each tooth in the three studied times, and the results of the Friedman test for comparison of the measurements with time (*p*< 0.05). For each tooth, means followed by same letter represents absence of statistical difference (*p*> 0.05).

Variable	Time	Mean	SD	p
Right central incisor	Initial (T_0_)	1.29^B^	0.47	0.037
	5 days (T_1_)	1.59^AB^	0.62	
	Final (T_2_)	1.71^A^	0.59	
Right premolar	Initial (T_0_)	1.62^B^	0.86	0.0344
	5 days (T_1_)	2.18^A^	0.81	
	Final (T_2_)	2.29^A^	0.99	
Right molar	Initial (T_0_)	2.06^B^	0.90	0.003
	5 days (T_1_)	2.71^A^	0.75	
	Final (T_2_)	2.97^A^	0.67	
Right lateral incisor	Initial (T_0_)	1.41	0.51	0.249
	5 days (T_1_)	1.59	0.51	
	Final (T_2_)	1.65	0.49	
Left central incisor	Initial (T_0_)	1.29	0.47	0.569
	5 days (T_1_)	1.41	0.51	
	Final (T_2_)	1.59	0.51	
Left premolar	Initial (T_0_)	1.76^B^	1.03	0.008
	5 days (T_1_)	2.24^AB^	0.97	
	Final (T_2_)	2.65^A^	0.93	
Left molar	Initial (T_0_)	2.41^B^	0.69	0.018
	5 days (T_1_)	2.62^A^	0.70	
	Final (T_2_)	3.26^A^	0.71	
Left lateral incisor	Initial (T_0_)	1.35	0.49	0.1171
	5 days (T_1_)	1.65	0.61	
	Final (T_2_)	1.76	0.56	

In relation to gingival recession, there was a statistical difference for the right and left premolar and molars (*p*<0.05). The recession in the premolars and molars significantly increased from T_0_ to T_2_ (Table 2).

**Table 2:** Evaluation of mean, standard deviation, median, minimum and maximum of the gingival recession of each tooth in the three studied times, and the results of the Friedman test for comparison of the measurements with time (*p*< 0.05)

Variable	Time	Mean	SD	p
Right central incisor	Initial	0.06	0.24	0.607
	5 days	0.00	0.00	
	Final	0.06	0.24	
Right premolar	Initial	0.38	0.78	0.036
	5 days	0.59	0.71	
	Final	0.71	0.85	
Right molar	Initial	0.65	0.79	0.001
	5 days	0.94	0.63	
	Final	1.26	0.66	
Right lateral incisor	Initial	0.00	0.00	___
	5 days	0.00	0.00	
	Final	0.00	0.00	
Left central incisor	Initial	0.00	0.00	___
	5 days	0.00	0.00	
	Final	0.00	0.00	
Left premolar	Initial	0.53	0.80	0.005
	5 days	0.71	0.92	
	Final	1.00	0.94	
Left molar	Initial	0.82	0.79	< 0.001
	5 days	0.79	0.73	
	Final	1.38	0.82	
Left lateral incisor	Initial	0.00	0.00	___
	5 days	0.00	0.00	
	Final	0.00	0.00	

The amount of attached gingiva decreased with time on the right premolar (*p*= 0.009) and the right (*p*= 0.006) and left molars (*p*= 0.002). The amount of attached gingiva decreased from T_0_ to T_1_ in the right premolar and right molar; and from T_0_ to T_2_ on the right premolar and right and left molar. However, from T_1_ to T_2_ the decrease was significant only on the left molar (*p*= 0.002) (Table 3). There were statistical significant differences in bleeding percentages from T_0_ to T_2_ in the buccal surface of the right central incisor and palatal left lateral incisor (Table 4).

**Table 3:** Evaluation of mean and standard deviation of the amount of attached gingiva of each tooth in the three studied times, and the results of the Friedman test for comparison of the measurements with time (*p*< 0.05). For each tooth, means followed by same letter represents absence of statistical difference (*p*> 0.05).

Variable	Time	Mean	SD	p
Right central incisor	Initial (T_0_)	3.53	1.70	0.174
	5 days (T_1_)	3.29	1.45	
	Final (T_2_)	3.29	1.61	
Right premolar	Initial (T_0_)	2.91^A^	1.63	0.009
	5 days (T_1_)	2.47^B^	1.56	
	Final (T_2_)	2.59^B^	1.55	
Right molar	Initial (T_0_)	3.06^A^	1.14	0.006
	5 days (T_1_)	2.44^B^	1.34	
	Final (T_2_)	2.29^B^	1.21	
Right lateral incisor	Initial (T_0_)	4.32	1.97	0.810
	5 days (T_1_)	4.26	1.77	
	Final (T_2_)	4.24	1.82	
Left central incisor	Initial (T_0_)	3.44	1.56	0.135
	5 days (T_1_)	3.50	1.75	
	Final (T_2_)	3.21	1.57	
Left premolar	Initial (T_0_)	2.44	1.41	0.070
	5 days (T_1_)	2.06	1.61	
	Final (T_2_)	1.82	1.66	
Left molar	Initial (T_0_)	2.71^A^	1.40	0.002
	5 days (T_1_)	2.56^A^	1.27	
	Final (T_2_)	2.00^B^	1.62	
Left lateral incisor	Initial (T_0_)	4.21	1.91	0.289
	5 days (T_1_)	4.00	1.54	
	Final (T_2_)	3.97	1.82	

**Table 4:** Evaluation of the frequency of bleeding among the three studied times. The statistical analyses showed that only for buccal right central incisor (*p*= 0.021) and palatal left lateral incisor (*p*= 0.008) the final measurement was higher than the initial.

Variable	Category	Initial (T_0_)		5 days (T_1_)		Final (T_2_)	
		Frequency	%	Frequency	%	Frequency	%
Buccal right central incisor	no	11	64.7	8	47.1	3	17.6
	yes	6	35.3	9	52.9	14	82.4
Buccal right premolar	no	9	52.9	8	47.1	7	41.2
	yes	8	47.1	9	52.9	10	58.8
Buccal right molar	no	10	58.8	11	64.7	7	41.2
	yes	7	41.2	6	35.3	10	58.8
Buccal right lateral incisor	no	11	64.7	11	64.7	8	47.1
	yes	6	35.3	6	35.3	9	52.9
Buccal left central incisor	no	7	41.2	7	41.2	4	23.5
	yes	10	58.8	10	58.8	13	76.5
Buccal left premolar	no	9	52.9	8	47.1	7	41.2
	yes	8	47.1	9	52.9	10	58.8
Buccal left molar	no	10	58.8	9	52.9	8	47.1
	yes	7	41.2	8	47.1	9	52.9
Buccal left lateral incisor	no	6	35.3	10	58.8	7	41.2
	yes	11	64.7	7	41.2	10	58.8
Palatal right central incisor	no	5	29.4	4	23.5	4	23.5
	yes	12	70.6	13	76.5	13	76.5
Palatal right premolar	no	10	58.8	9	52.9	4	23.5
	yes	7	41.2	8	47.1	13	76.5
Palatal right molar	no	9	52.9	8	47.1	6	35.3
	yes	8	47.1	9	52.9	11	64.7
Palatal right lateral incisor	no	7	41.2	6	35.3	4	23.5
	yes	10	58.8	11	64.7	13	76.5
Palatal left central incisor	no	7	41.2	5	29.4	4	23.5
	yes	10	58.8	12	70.6	13	76.5
Palatal left premolar	no	7	41.2	7	41.2	5	29.4
	yes	10	58.8	10	58.8	12	70.6
Palatal left molar	no	10	58.8	9	52.9	5	29.4
	yes	7	41.2	8	47.1	12	70.6
Palatal left lateral incisor	no	9	52.9	3	17.6	1	5.9
	yes	8	47.1	14	82.4	16	94.1
Total		17	100	17	100	17	100

## DISCUSSION

Although there are few studies that relate periodontal status and SARME, the complications described in these studies include osseous defects, reduction of the interproximal papilla and gingival recession.^9^ ^,^ ^17^ To minimize these effects, proper planning, adequate surgical technique, maintenance of osseous tissue in mesial part of the roots of central incisors and preservation of gingiva are necessary.^20^ ^-^ ^23^

The first premolars and molars were chosen because they are the teeth supporting the expansion appliance and therefore, they were subjected to the influence of the forces exerted by the appliance. The maxillary central incisors, the teeth located near the area of the interdental osteotomy, can be affected by the fracture and by the activation of the expander screw. The maxillary lateral incisors were chosen as a control due to their distance from the supporting teeth and the interdental osteotomy area, and because they do not suffer from the direct action of the expansion appliance. The maxillary second premolars, canines and second molars were not used because they are affected by the action of the expansion appliance due to the lingual bar connecting the first premolars to the first molars and due to their proximity of the supporting teeth, respectively.

In the present study, the statistically significant increase of CAL of the right central incisors in all studied times is due to the surgical procedure of SARME. Otherwise, in the right lateral incisor there was no significant increase. In addition also the right and left premolar had a statistically significant increase in CAL. Landes et al^2^ observed that the greater osseous resorption on the buccal surface of the first premolars found in patients submitted to SARME with dental-supported appliances was due to the anatomical location in a maxillary area where the hard palate is narrower cranially. In this area, buccal movements can cause fenestrations or osseous dehiscence. In the present study, the first molars showed statistically significant increase in CAL. Maybe in molar and premolar areas the increase of CAL is related to the association between the appliance forces and the surgical trauma, instead of the anterior teeth where the increase of CAL might be related only to surgical trauma.^24^ ^,^ ^25^

The observation of the right side presenting a greater CAL difference than the left side may be accounted for the fact that the patients were right handed. Individuals who are more dexterous with their right hands initiate the brushing by the left side, and therefore, this side is more effective, since the brushing in the right side is reverse and the handle of the brush is more difficult.^26^ As a result, the bacterial plaque on the buccal surface of teeth in the maxillary arch is greater on the right side than the left.^27^

The CAL increased at T_2_ in relation to the earlier evaluated times because the appliance makes brushing more difficult. The increase in the probing depth on the buccal surface of the teeth can be attributed to worse brushing quality after the surgery due to the greater sensitivity during this period. The palatal surface of the anterior teeth is less sensitive in the postsurgical period, and thus, brushing is performed more efficiently. 

Though the orthodontic bands are well adapted to molars and premolars, when the appliance is removed, gingival inflammation is observed in this region. Thus, the probing depths in these areas were statistically significant at each evaluation. This is in agreement with the results of other studies.^28^

The supporting teeth had statistically significant changes in CAL. There are two possible explanations for this finding: first, even with the release of the areas of maxillary resistance with SARME, when the appliance is activated, the supporting teeth are buccally inclined,^16^ possibly causing a decrease in the thickness of the buccal cortical bone and the appearance of osseous dehiscences,^29^ leading to gingival recession in these teeth; second, the insertion of the orthodontic bands next to the sulcus facilitates the retention of bacterial plaque in this area. Most of the gingival recession observed in the molars was significantly different between T_0_ and T_2_ and between T_1_ and T_2_ because this period included the activation of the appliance and the retention, and, if teeth were buccally inclined during the expansion, they were maintained in this position for six months, causing the recession. The period between T_0_ and T_1_ was too short for the formation of gingival recession. Central and lateral incisors did not present gingival recession because they were neither buccally inclined after activation of the appliance, nor did they receive orthodontic bands.

Another important aspect of the periodontal evaluation is the analysis of the amount of attached gingiva. The loss of attached gingiva as a result of the osteotomy is a risk that should be considered.^9^ A decrease in the amount of attached gingiva can be related to the increase in gingival recession or to the increase in probing depth. 

The occurrence of bleeding is an indication of gingival inflammation.^30^ In the present study, there was an increase of the frequency of gingival bleeding in all teeth over time except on the buccal surface of the left lateral incisor 

## FINAL CONSIDERATIONS

The results obtained in this study indicate that SARME can cause alterations in the gingival tissues of patients, and therefore must be performed with appropriate technique and careful manipulation of the gingival tissues. An option to try to minimize these effects would be the modification of the anchor protocol of the expansion appliance, performing SARME with devices supported on temporary anchorage devices (TADs), so that the effects on the anchoring teeth are reduced. 

## CONCLUSIONS

1. There was a statistically significant difference in the CAL for the following teeth: the right central incisor, right and left premolars, and right and left molar.
2. The teeth supporting the expansion appliance, the first premolars and molars, showed statistically significant gingival recession over time.
3. The amount of attached gingiva decreased significantly for right and left molars and for right premolars over the evaluated time.
4. There was an increase in the frequency of gingival bleeding on all surfaces of all teeth over time, except on the buccal surface of the left lateral incisor.
